# Management of retroperitoneal fibrosis with endovascular aneurysm repair in patients refractory to medical management

**DOI:** 10.3389/fsurg.2022.946675

**Published:** 2022-07-15

**Authors:** Sherif Sultan, Yogesh Acharya, Mohieldin Hezima, Joshua Ramjohn, David Miresse, Keegan Chua Vi Long, Osama Soliman, Niamh Hynes

**Affiliations:** ^1^ Western Vascular Institute, Department of Vascular and Endovascular Surgery, University Hospital Galway, National University of Ireland, Galway, Ireland; ^2^ Galway Clinic, Royal College of Surgeons in Ireland and National University of Ireland, Galway affiliated Hospital, Doughiska, Ireland; ^3^ CÚRAM-CORRIB-Vascular Group, National University of Ireland, Galway, Ireland

**Keywords:** retroperitoneal fibrosis, immunoglobulin G4-related disease, medical management, endovascular procedures, endovascular aneurysm repair

## Abstract

**Background:**

Early diagnosis and treatment of under-recognized retroperitoneal fibrosis (RPF) are essential before reaching the poorly responsive fibrotic stage. Although most patients respond to medical therapy, relapses and unresponsiveness are common. However, open surgery in medically resistant patients is associated with major adverse clinical events.

**Methods:**

This is a single-centre longitudinal study of optimal medical therapy (OMT) vs. endovascular aneurysm repair (EVAR) in patients presenting with RPF to our tertiary referral vascular centre. Out of 22,349 aortic referrals, we performed 1,555 aortic interventions over twenty years. Amongst them, 1,006 were EVAR, TEVAR and BEVAR. Seventeen patients (1.09%) had documented peri-aortic RPF.

**Results:**

Out of the 17 RPF patients, 11 received OMT only, while 6 underwent EVAR after the failure of OMT. 82% (*n* = 14) were male, and the median follow-up was 62.7 months (IQR: 28.2–106). Nine (52%) had immunoglobulin G4-related disease (4 OMT vs. 5 EVAR). EVAR patients had 100% technical success without perioperative mortality. Furthermore, all the EVAR patients were symptom-free following the intervention. Pre-operative aortic RPF index (maximum peri-aortic soft tissue diameter/maximum aortic diameter) was higher in the EVAR than in OMT. However, there was a significant decrement in the aortic RPF index following EVAR (*P *= 0.04).

**Conclusion:**

We believe that when optimal medical therapy fails in RPF, EVAR provides a promising outcome. Further studies are recommended to establish the role of endovascular repair.

## Introduction

Retroperitoneal fibrosis (RPF) is an uncommon disorder that causes fibrosis and scarring around the retroperitoneal space with resultant complications like ureteral obstruction and periaortitis (1–4). RPF starts as a mild inflammation around the infrarenal aorta with adventitial and periadventitial inflammation, medial thinning, and a chronic retroperitoneal inflammatory process (3–9). National Organization for Rare Diseases (NORD) (8) has stated that the exact cause of this condition is unknown in about two-thirds of cases. RPF typically develops in late middle age, i.e., 40–60 years, twice to three times more often in men than women (1–9).

Immunoglobulin G4-related disease (IgG4-RD) is a secondary RPF variant that constitutes histological predominance of lymphocytes and plasma cells (3, 4). IgG4-related RPF often has some response to glucocorticoid therapy; however, if misdiagnosed as retroperitoneal visceral malignancy, it will result in unnecessary surgical intervention (3–9). Early detection, accurate diagnosis and treatment are imperative (10–13).

Aortic antigenic targets and antibodies directed against aortic endothelial cells in RPF start as a local inflammatory response to atherosclerotic plaque antigens, leading to a local autoimmune process and cardiovascular inflammation. This results in vascular dysfunction by inducing the expression of endothelial adhesion molecules, cytokine production, and apoptosis (4, 7, 9, 13). We believe that endovascular exclusion of the infrarenal aortic wall from the circulation will cease these cascades of reactions, resulting in modulation and healing (11). Despite open surgery traditionally being the only option for patients refractory to medical management, advances in medicine have allowed us to employ endovascular repair in patients with RFP. However, we reserved it for patients who did not show a favourable response with medical management alone. Therefore, in this study, we aim to compare the outcomes of endovascular repair in RPF patients who were refractory to the optimal medical management.

## Materials and methods

This is a single-centre longitudinal study of optical medical management and endovascular aneurysm repair (EVAR) in patients referred to our tertiary vascular centre with RPF from December 2002 to 2020. We included the patients with established RPF on imaging modalities. Patients with a history of tuberculosis, actinomycosis, histoplasmosis, recent trauma, illicit substance abuse, and inflammatory AAA were excluded.

The primary outcome is symptom-free survival. The secondary outcomes are all-cause mortality, perioperative mortality and technical success. Technical success is the successful deployment of the EVAR device without peri-procedural complications, like surgical conversion or death, and ELs (type I or III) or graft obstruction, kinks or twists.

### Patients

All our patients were fully worked up by rheumatologists, nephrologists, urologists, and immunologists before being referred to us. They all had targeted computerized tomography (CT) and/or magnetic resonance imaging (MRI) scans, duplex ultrasound scanning (DUS), routine blood tests, renal function tests, and biopsies as necessary. Furthermore, optimal medical treatment had already been initiated before the referral.

All of our patients complained of a dull pain in the abdomen and lower back that is hard to pinpoint with swelling and discolouration of both legs and a dragging sensation in the scrotum. Patients had nausea, vomiting, loss of appetite, weight loss, and felt thirstier than usual, even in the absence of diabetes mellitus. None of our patients had a history of tuberculosis, actinomycosis, histoplasmosis, or recent trauma. All of our patients denied using cocaine and any other illegal substances.

**Figure 1:**
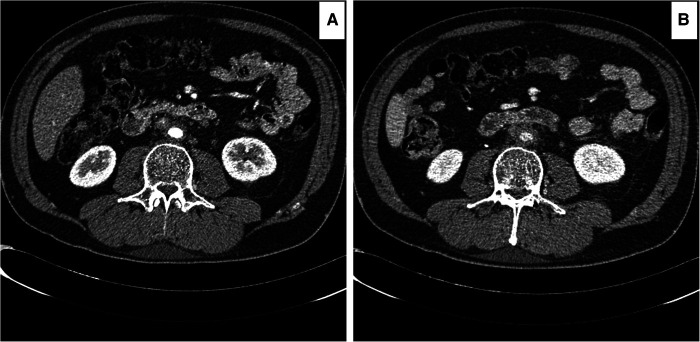
Computed tomography angiography (CTA) images showing worsening of the peri-aortic soft tissue after medical management. (**A**) RPF with less than 3 mm enhancements around the aorta and 21 mm aortic diameter. (**B**) after six months of steroid and mycophenolate, the enhancements increased to 5 mm, and the aortic diameter decreased to 18 mm. The patient had continuous abdominal and low back pain, not responding to the WHO one and two pain ladder medications.

Our overall management goal was to relieve ureteric obstruction, decrease peri-aortic fibrosis, and abolish pain. For at least three months, all our patients received optimal medical therapy (OMT) with steroids and/or mycophenolate mofetil. Patients who did not benefit from OMT and had failed medical management with anti-inflammatory medications, corticosteroids or immunosuppressants (mycophenolate mofetil) were labelled medically resistant and were offered EVAR (Figure 1).

**Figure 2:**
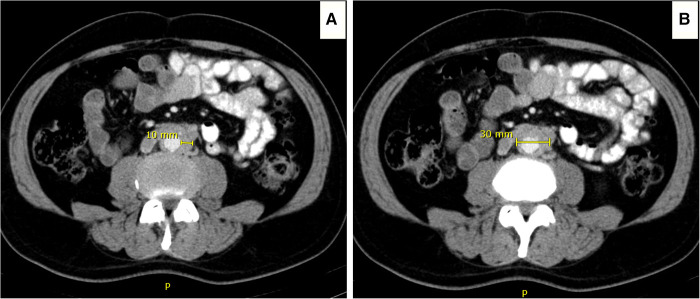
Computed tomography angiography (CTA) images illustrating the determination of the aortic retroperitoneal fibrosis index (peri-aortic soft tissue enhancement over maximum aortic diameter). (**A**) Peri-aortic soft tissue enhancement of 10 mm. (**B**) overall aortic enhancement of 30 mm, including the peri-aortic soft tissue enhancement.

All our patients had a targeted computed tomography angiography (CTA) initially and post-management, which was used to calculate the aortic RPF indices. Aortic RPF index was defined as maximum peri-aortic soft tissue diameter relative to maximum aortic diameter. These measurements were taken after defining the aortic centre-line on arterial phase images. Aortic RPF index is zero for patients without RPF or any enhancement of peri-aortic soft tissue. Furthermore, we approximate the cross-sectional area of the peri-aortic soft tissue enhancements by using the formula π (Pi) times the radius squared (π * r^2^) (cross-sectional area of periaortic soft tissue enhancement = cross sectional area of the total aortic enhancement including aorta - cross sectional area surface area of aorta) (Figure 2).

### Follow-up

The patients were regularly followed through thorough clinical evaluations, DUS, and CTA. We performed CTA initially, post-EVAR, at nine months, and annually after that. However, DUS was conducted at six weeks, six months, and every nine months.

### Statistical analysis

Jamovi (the Jamovi project 2021, version 1.6) was used for statistical analyses. We summarized continuous outcomes through mean value supported with standard deviation and median value supported with interquartile range as necessary. Similarly, the categorical outcome was summarized with percentages and/or proportions. We employed Wilcoxon signed-rank test or Fisher exact test for statistical significance with *P* < 0.05 as statistically significant.

## Results

Out of 22,349 aortic referrals to our tertiary referral centre, we performed 1,555 aortic interventions over twenty years. Amongst them, 910 were EVAR ± iliac branch devices (IBD), and 96 were thoracic endovascular aortic repair (TEVAR)/branched endovascular aortic repair (BEVAR).

**Figure 3:**
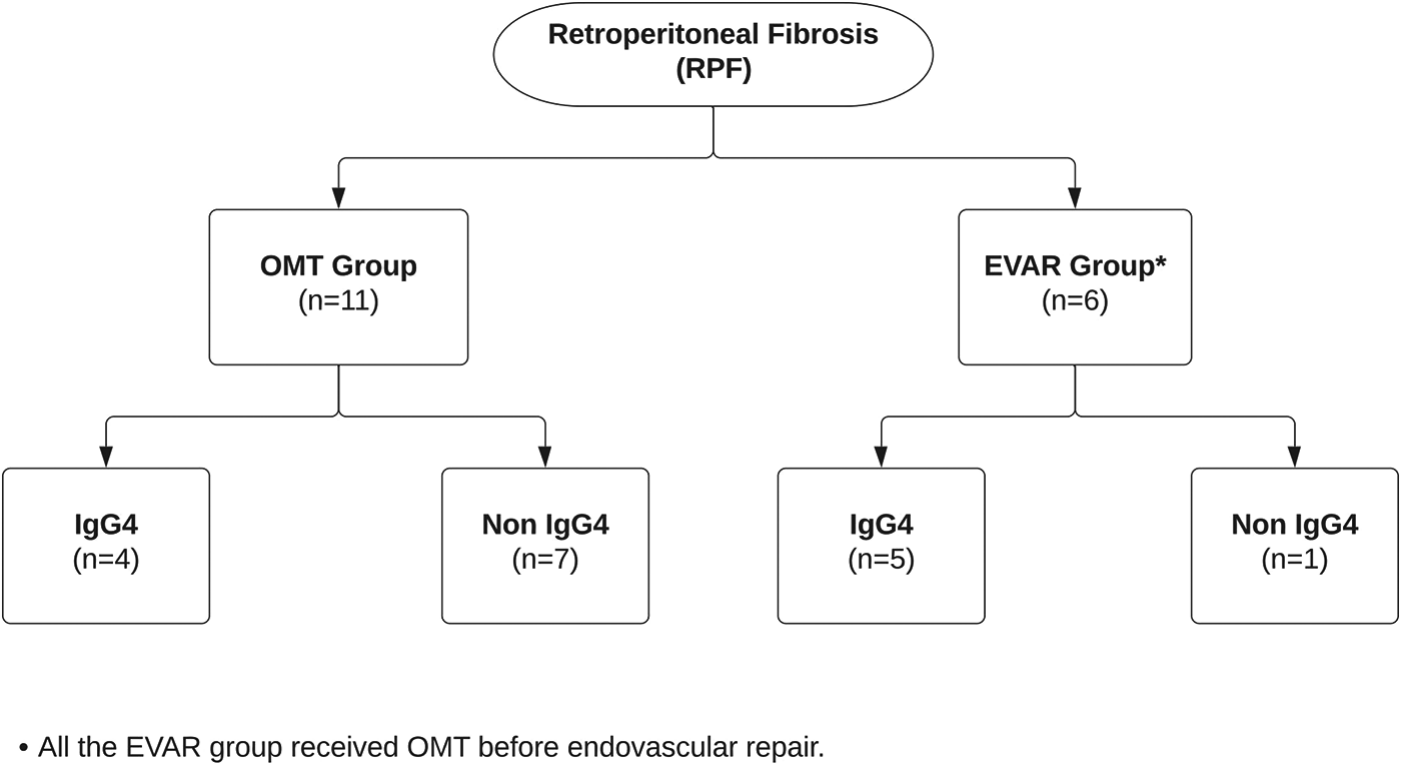
Flowchart showing patients with retroperitoneal fibrosis who were managed with optimal medical therapy vs. endovascular aneurysm repair.

Over the past two decades, 17 patients (1.09%) were referred to our vascular service with RPF. Amongst them, six patients (6/17) underwent EVAR following a deterioration despite optimal medical management. Nine patients (9/17) were IgG4-RD positive, of which five patients were medically resistant and were offered EVAR. Eight patients (8/17) were IgG4-RD negative, of which one was offered EVAR due to OMT failure (Figure 3). All medically resistant patients were referred to our services after more than a year post-RPF diagnosis. The baseline demographics of these patients are given in Table 1.

**Table 1:** Baseline demographics of the patients.

	Retro-peritoneal Fibrosis (*N* = 17)		*P*-value
	Optimal Medical Therapy (OMT) Only (*n* = 11)	Endovascular Aneurysm Repair (EVAR) (*n* = 6)	
Time from diagnosis to vascular referral	6–11 months	14–22 months	0.01^*^
Male	9	5	1.00
Age (years), Mean ± Standard Deviations (range)	66.6 ± 12.9 (50–88)	64.0 ± 7.01 (57–76)	0.76
Smokers	4	4	0.33
Diabetes Mellitus (DM)	2	2	0.58
Chronic Obstructive Pulmonary Disease (COPD)	1	1	1.00
Hypercholesterolemia	3	3	0.60
Hypertension (HTN)	3	3	0.60
Ischemic Heart Disease (IHD)	2	3	0.28
Chronic Renal Failure (CRF)	0	3	0.02
Peripheral Vascular Disease (PVD)	1	3	0.09
Thyroid Disease	1	3	0.09
Depression	2	2	0.58
Diverticulosis	2	2	0.58
Stroke	1	0	1.00
Malignancy	5	3	1.00

* *Significant.*

All of our patients had elevated C-reactive protein; however, none developed post-implantation inflammatory syndrome post-EVAR (Table 2). Following EVAR, all of our patients had improvements in the biochemical parameters. We had only one patient who was anti-nuclear antibody (ANA) positive. This patient underwent EVAR after the failure of medical management.

**Table 2:** Laboratory and biochemical parameters of the retroperitoneal fibrosis patients at the initial diagnosis, post optical medical treatment (OMT), and post endovascular aneurysm repair (EVAR).

Laboratory values	Groups	Initial (at presentation)	Post-management (at last follow-up)	*P*-value
C-Reactive Protein (CRP), mg/L mean ± SD (range)	Overall	50.90 ± 81.7 (0.60–281)	19.90 ± 28.40 (0.80–84.0)	**0**.**19**
	EVAR	47.10 ± 60.80 (4.50–148)	29.70 ± 34.40 (1.80–84.0)	**0**.**59**
	OMT	53.20 ± 96.50 (0.60–281)	14.60 ± 24.80 (0.80–84.0)	**0**.**26**
White cell count (WCC), 10^9^/L mean ± SD (range)	Overall	11.14 ± 7.18 (5.10–32.9)	8.16 ± 3.46 (2.60–15.6)	**0**.**17**
	EVAR	8.28 ± 1.58 (5.10–9.10)	6.98 ± 1.03 (5.90–8.30)	**0**.**16**
	OMT	11.70 ± 8.64 (6.10–32.9)	8.80 ± 4.17 (2.60–15.6)	**0**.**37**
Neutrophils, 10^9^/L mean ± SD (range)	Overall	6.72 ± 6.95 (2.20–30.2)	5.72 ± 2.74 (1.27–11.4)	**0**.**62**
	EVAR	4.16 ± 1.50 (2.20–6.20)	4.85 ± 1.00 (3.80–6.20)	**0**.**41**
	OMT	8.14 ± 8.42 (3.90–30.2)	5.00 ± 3.29 (1.27–11.4)	**0**.**31**
Eosinophil, 10^9^/L mean ± SD (range)	Overall	0.29 ± 0.26 (0.00–1.00)	0.27 ± 0.23 (0.00–1.00)	**0**.**83**
	EVAR	0.28 ± 0.08 (0.20–0.40)	0.29 ± 0.11 (0.19–0.50)	**0**.**87**
	OMT	0.30 ± 0.33 (0.00–1.00)	0.29 ± 0.24 (0.00–1.00)	**0**.**94**
Estimated glomerular filtration rate (eGFR), ml/min mean ± SD (range)	Overall	69.50 ± 17.30 (20–90)	64.40 ± 22.8 (15–90)	**0**.**51**
	EVAR	64.40 ± 28.10 (20–90)	63.80 ± 30.60 (15–90)	**0**.**97**
	OMT	72.10 ± 9.65 (55–82)	64.70 ± 18.60 (32–90)	**0**.**27**

*EVAR, endovascular aneurysm repair; OMT, optimal medical therapy; SD, standard deviation.*

**Figure 4:**
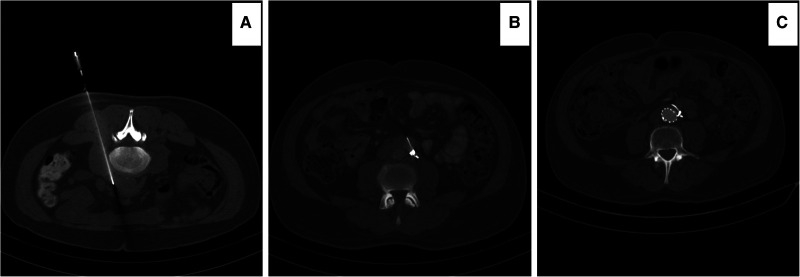
Computed tomography angiography (CTA) images illustrating: (**A**) percutaneous computed tomography (CT) scan guided biopsy of the retroperitoneal tissue in a prone patient. The patient developed bleeding during the biopsy. (**B**) the patient required three COOK coils (COOK medical LLC, Bloomington, IN) to stop the bleeding. (**C**) The patient was medically resistant and required EVAR 14 months post diagnosis. Note that the coils are now in the retroperitoneal space as the enhancements of the retroperitoneal fibrous tissue disappeared post-EVAR.

Most (*n* = 5) EVAR cases were performed using AFX (Endologix, Irvine, CA) and one with FORTON (Cordis, Miami Lakes, FL). We opted for AFX as it is an endoskeleton unibody with ultrathin ePTFE and depends on anatomical fixation over the aortic bifurcation rather than radial force. However, we supplemented the distal end with Bentley (Bentley InnoMed GmbH, Hechingen, Germany) covered stent graft. Also, it could be applied in narrow distal aortic diameter. Five of our six patients had distal aortic diameter <14 mm, which precludes any other bi-iliac aortic graft in the market. The patient treated with FORTON Cordis in 2003 was the only non-IgG4-RD RPF treated by EVAR. Amongst EVAR, five patients had aortic biopsies; however, three of them ended with bleeding complications that required embolization by coils to the site of biopsy (Figure 4).

### Symptom-free survival

**Figure 5:**
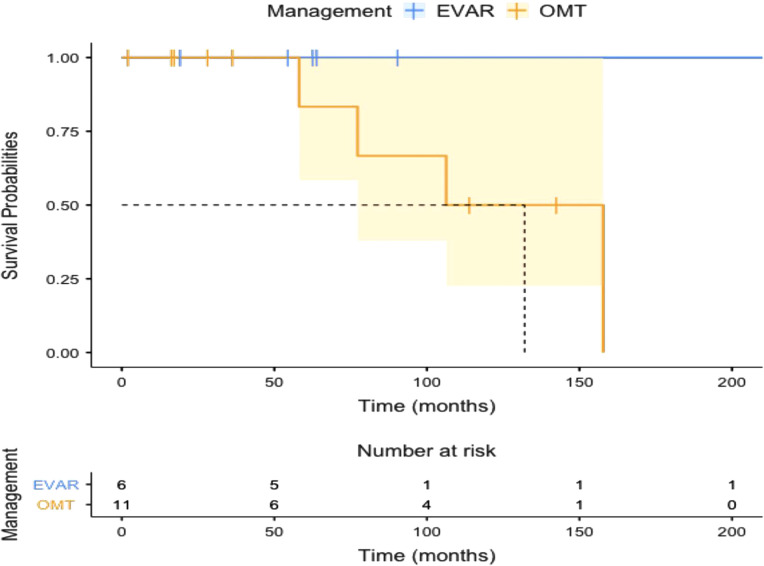
Kaplan-Meier plot showing symptom-free survival in patients with optimal medical therapy (OMT) and endovascular aneurysm repair (EVAR).

All the patients who underwent EVAR had symptom-free survival following the procedure throughout follow-up. The Kaplan-Meier plot for symptom-free survival is given in Figure 5 (log-rank test: *χ*² = 2.25; *P* = 0.13). All EVAR patients went off all of the painkillers.

### Peri-operative and all-cause mortality

**Figure 6:**
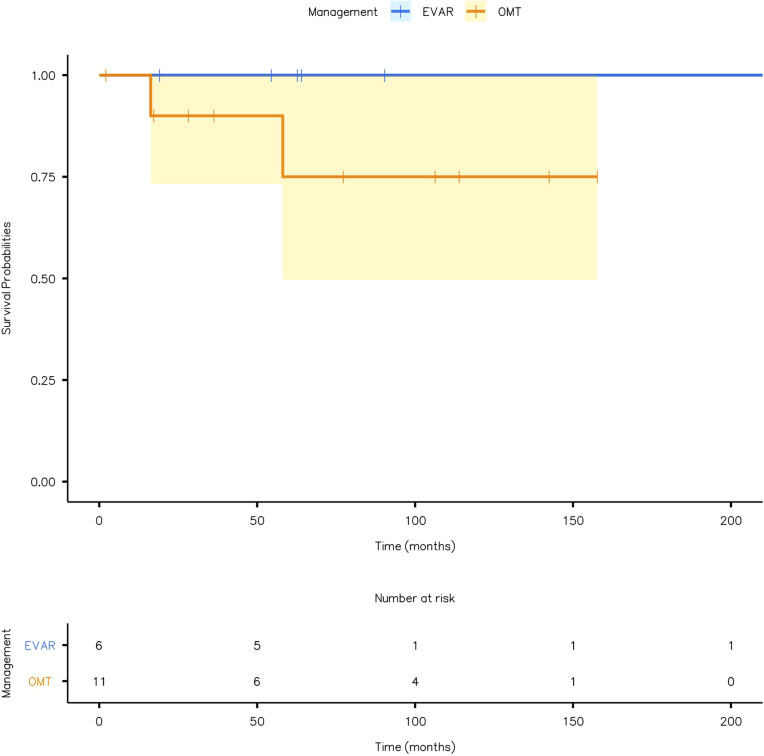
Kaplan-Meier plot showing all-cause mortality in patients with optimal medical therapy (OMT) and endovascular aneurysm repair (EVAR).

Kaplan-Meier survival plot for all-cause mortality is given in Figure 6 (log-rank test: *χ*² = 1.27; *P* = 0.26). None of the patients had peri-operative mortality post-EVAR. The OMT group suffered two mortalities during follow-up, one with renal impairment and the other with acute pneumonia after COVID-19.

### Aortic RPF index

The aortic RPF indices and cross-sectional area of the peri-aortic soft tissue are given in Table 3. Overall, the EVAR group had a higher initial aortic RPF index than the OMT group (*P* = 0.01).

**Table 3:** Retro-peritoneal fibrosis (RPF) aortic index and cross-sectional area of the peri-aortic soft tissue enhancements at the initial diagnosis, post optical medical treatment (OMT) and post endovascular aneurysm repair (EVAR).

	Aortic RPF Index (Mean ± SD, range)			Cross-sectional area mm^2^ (Mean ± SD, range)		
	EVAR Group (*N* = 6)	OMT Group (*N* = 11)	*P*-value	EVAR Group (*N* = 6)	OMT Group (*N* = 11)	*P*-value
Initial (at presentation)	0.36 ± 0.07 (0.27–0.49)	0.24 ± 0.08 (0.12–0.37)	0.01^*^	1182 ± 537 (628–2177)	650 ± 476 (207 –1627)	0.05^*^
Post-OMT	0.35 ± 0.06 (0.27–0.42)	0.18 ± 0.10 (0.00–0.37)	0.01^*^	1069 ± 638 (374–2177)	542 ± 476 (0–1627)	0.07
Post-EVAR	0.21 ± 0.14 (0.04–0.36)	–	–	803 ± 833 (111–2177)	–	–
Post-EVAR vs. Post-OMT	0.21 ± 0.14 (0.04–0.36)	0.18 ± 0.10 (0.00–0.37)	0.59	803 ± 833 (111–2177)	542 ± 476 (0–1627)	0.42

* *Significant.*

**Figure 7:**
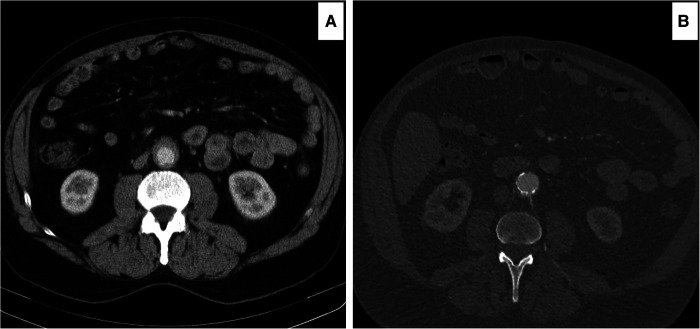
Computed tomography angiography (CTA) images showing decrement of the peri-aortic soft tissue enhancements following medical management. (**A**) Initial 4 mm peri-aortic soft tissue enhancement. (**B**) Two years post medical management, the peri-aortic soft tissue enhancement was reduced to 1 mm.

**Figure 8:**
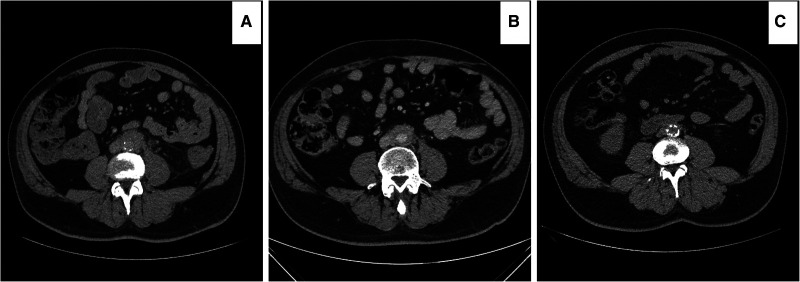
Computed tomography angiography (CTA) images showing improvement of the peri-aortic soft tissue enhancements following endovascular aneurysm repair (EVAR). (**A**) Initial peri-aortic soft tissue enhancement of 6 mm. (**B**) failure of medical treatment at 15 months with an enhancement increment to 8 mm. (**C**) Nine months post-EVAR, enhancements of 2 mm. All the symptoms and signs disappeared, and the patient is living a symptom-free life.

**Figure 9:**
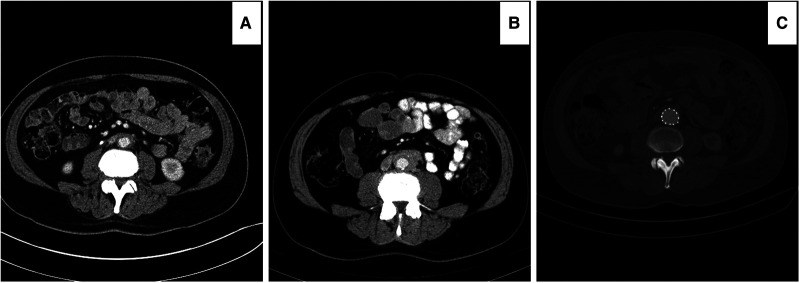
Computed tomography angiography (CTA) images showing improvement in peri-aortic soft tissue enhancement following endovascular aneurysm repair (EVAR): (**A**) peri-aortic enhancement of 3 mm on optimal medical therapy for three months. (**B**) Peri-aortic enhancements increased to 6 mm after optimal medical management for nine months. (**C**) peri-aortic enhancements disappeared with no evidence of RPF six months post-EVAR.

Overall, there was a non-significant decrement in the aortic index from the initial diagnosis to post three to six months of the OMT in both the medical (*P* = 0.14) and EVAR (*P* = 0.72) groups (Figure 7). However, the aortic RPF index decreased significantly post-EVAR (*P* = 0.04) (mean follow-up of 84.80 ± 69.30 months) (Figures 8, 9). The pre-operative cross-sectional periaortic soft tissue area was almost double in the EVAR group compared to the OMT group. Medically resistant patients with EVAR had their cross-sectional periaortic soft tissue area decreased by nearly two-thirds within six months and reached near-normal within 24 months of follow-up.

### Follow-up

Median follow-up duration was 62.7 months (IQR: 28.2–106); EVAR 63.4 months (IQR: 56.5–83.8) vs. OMT 58.1 months (IQR: 22.7–110).

## Discussion

Two-thirds of RPF are idiopathic. Twelve per cent are secondary to the use of ergot alkaloid derivatives. However, ten per cent are associated with malignancies, including lymphoma, retroperitoneal sarcoma, carcinoid tumour, thyroid neoplasms, or metastatic gastrointestinal tumours (14–18).

Idiopathic and benign forms of RPF have a good outcome, whereas RPF secondary to malignancy has a poor prognosis (19). Therefore, the most crucial challenge is distinguishing benign from malignant RPF at imaging.

We have a simple algorithm that we usually follow. If aorta and inferior vena cava (IVC) is anteriorly displaced with posteriorly enlarged lymph nodes to the great vessels, in a more cephalic location in the retroperitoneum, it is usually a malignant RPF, and it often exerts mass effect on neighbouring structures (14). Benign RPF soft-tissue mass always spares the posterior aspect of the great vessels and does not cause vascular displacement. It is located mainly distal to the renal hilum (20) and has an infiltrative aspect enveloping rather than displacing adjacent structures (14). In this aspect, most physicians relied on complex surgery to relieve the obstruction caused by RPF, with an adjuvant of double J ureteric stents followed by open or laparoscopic ureterolysis. Such an approach does not address systemic symptoms, such as pain, weight loss and anaemia, or the disease's underlying causes - inflammation and fibrosis.

Although the pathophysiology of this process is unknown, an exaggerated local inflammatory response to oxidized low-density lipoprotein in aortic plaque has been postulated. Treatment for IgG4-RD includes high-dose steroids and Disease-Modifying Anti-Rheumatic Drugs (DMARDs); rituximab, a monoclonal antibody targeting B cells, tapering off over six months (4–7). Maintenance therapy with prednisone is recommended for up to three years. The disease recurs in 30%, and the use of mycophenolate mofetil, tamoxifen, or methotrexate should be considered for these patients.

**Figure 10:**
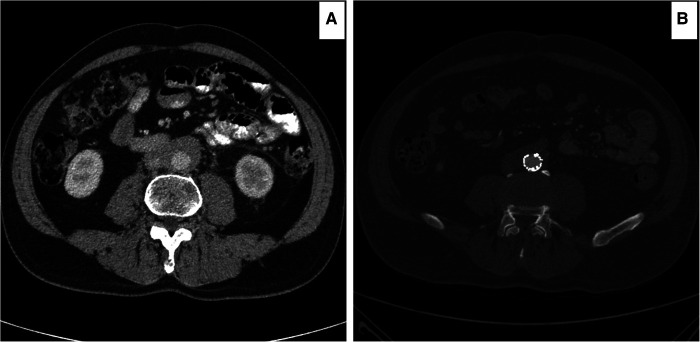
Computed tomography angiography (CTA) images showing improvement of the peri-aortic soft tissue enhancements following endovascular aneurysm repair (EVAR). (**A**) Post 16 months on optimal medical therapy, the peri-aortic soft tissue enhancement of 11 mm. (**B**) One year post-EVAR, all symptoms and signs disappeared with peri-aortic soft tissue enhancement of 3mm.

All 17 patients were primarily managed with a minimum of three months of steroids. Symptoms and signs initially improved. But six patients became refractory to all medical therapy modalities, with flared up abdominal pain and excoriating back pain demanding an endovascular intervention by EVAR. Therefore, OMT was only successful in 11 patients in our study. The other six medically resistant patients, who required EVAR, were referred to our services more than a year after diagnosis. This may explain why their indices were higher than OMT. In those treated by EVAR, the aortic index dropped significantly after excluding the infra renal aortic wall from the circulation (Figure 10). Comparing post-EVAR to OMT, the aortic RPF indices indicate that EVAR was as useful as OMT in decreasing the peri-aortic soft tissue enhancements.

The aortic RPF index fell by 80% in all EVAR patients and was back to normal in 66.66% of the EVAR managed patients. In patients with IgG4-related periaortitis who were responsive to OMT, their aortic RPF index dropped in 20% of the patients.

In all EVAR patients, CTA demonstrated suppression of aortic inflammation and complete aortic remodelling within six months, and all patients went off their analgesia and immune modulations drugs. All OMT patients were kept on DMARDs lifelong.

Our management aim in RPF is to relieve clinical symptoms of the disease and abolish pain through the prevention and management of the fibrotic process. The fundamental goal is to release the encapsulated structures around the aorto-iliac, including the IVC and ureters.

IgG4-related aortic lesions are difficult to distinguish from aortitis, peri-aortitis, inflammatory AAA and RPF (21). Corticosteroid therapy's effectiveness for IgG4-related aortic lesions remains controversial as it may fail in modulating the dense periaortic fibrous tissues (9, 22). A biopsy must be done to clarify the diagnosis and rule out malignancies or infectious diseases in cases where aortitis is refractory to optimal medical therapy.

The endovascular management of complicated peri-aortic inflammation is a challenging task, and there are no fixed guidelines or algorithms to follow after medical management failure. EVAR attenuates proinflammatory T-cell changes compared with open repair. T-cell activation reduction with impaired responsiveness to superantigen (11) implies that the immunological sequelae of EVAR for IgG4 aortitis are more favourable than after the open approach, with potentially less risk of adverse outcomes.

The aortic RPF index increases with increasing peri-aortic enhancements in RPF. Monitoring with inflammatory markers and CT scan should be continued every three months while on treatment and every six months when off treatment, as RPF has recurred in some cases, even years after treatment. Ureter obstruction recurs in about half of all people who had surgery.

Our six EVAR patients had failed a nonsurgical RPF approach with two drugs - prednisone and/or mycophenolate mofetil (MMF). As their systemic symptoms did not improve, an innovative approach with EVAR was warranted. The orthodox approach in an IgG4-related inflammatory AAA is bilateral ureterolysis through an open surgical approach. However, bilateral ureterolysis through an open surgical approach in an IgG4-related inflammatory AAA is associated with high morbidity. EVAR will avoid extensive dissection, thus minimizing the risk of morbidity and mortality.

Our results contradict the finding of Scheel (23) from John Hopkins, who used the prednisone/mycophenolate mofetil combination therapy to treat RPF patients. They reported a response rate of 95 per cent and a recurrence rate of 5 per cent. More than one-third (35%) were resistant to OMT in our study, all requiring EVAR to achieve a better clinical outcome. However, these are only the percentage of the patients who were referred or presented to us with RPF.

Our results mirror the findings of Ikeda et al. (24), who used EVAR to treat IgG4-related peri-aortitis, with a one-year follow-up that revealed complete resolution of periaortic inflammation. EVAR is best suited for an IgG4-related inflammatory AAA as the actual luminal diameter of peri-aortitis is near normal. This was depicted by the RPF aortic index, which returned to a near-normal level after EVAR. However, the thickened, diseased, disrupted aortic wall can induce a false aneurysm (25).

Our cases supplement previous publications on EVAR for complicated infrarenal peri-aortic RPF due to IgG4-related peri-aortitis in high-risk patients with ureteric compression or obstruction. In previous studies, the solution was to relieve the symptoms with ureteric stents or ureterolysis rather than treat the underlying cause (26, 27). Our results complement the finding of Hapka et al. (27), who employed EVAR when RPF was associated with hydronephrosis, common iliac artery (CIA) stenosis and saccular aneurysm.

Kawashima et al. (28) confirmed interleukin (IL) 6 upregulation in the adventitia of activated immune reactions in IgG4-aortic aneurysms (AA) patients. OMT regimens, including tocilizumab, a human monoclonal antibody that competitively inhibits IL-6 binding to its receptor for refractory disease IgG4-AA patients, are appropriate adjuvant to steroids. Furthermore, it could serve as a new effective therapy for IgG4-AAs (29). Our six patients received tocilizumab, but only three improved.

Surprisingly, expert consensus on initiating treatment in IgG4-RD active disease patients is low, considering irreversible damage to visceral organs may happen within weeks. The strategy and sole aim of management is avoidance of fibrosis and its potentially devastating impact on organs. RPF response is not sustained if glucocorticoids are decreased (30). However, remission induction and maintenance differ from country to country, depending on B-cell depletion therapy availability. Japanese rely upon glucocorticoid monotherapy. In contrast, North Americans and Europeans emphasize the early introduction of glucocorticoid-sparing agents, including B cell–depleting strategies (30).

There was no need for remission induction and maintenance following EVAR for our six patients. The physician must initiate lateral thinking, as minimally invasive infra-renal aortic exclusion by stent graft may be an ideal simple solution. Early diagnosis and treatment of the under-recognized RPF/IgG4-related disease are important before reaching the poorly responsive fibrotic stage with morbidity related to organ damage. Although most patients respond to medical therapy, relapses are still common. Inflammatory aortic aneurysms patients behave worse than patients with noninflammatory aortic aneurysms (12). Preoperative suspicion and the endovascular option offer superior results for challenging and complex aortic pathologies. However, our study is limited due to the relatively small number of patients owing to an uncommon condition and its retrospective nature. Furthermore, we compared two different management strategies, medical vs. surgical, in medically resistant patients, which may represent a source of bias.

## Conclusion

EVAR in RPF medical resistant patients is a valuable option in the armamentarium of nephrologists and rheumatologists. An endovascular intervention provides a promising outcome in RPF/IgG4-RD periaortitis, which is refractory to medical therapy. It is safe and easy to deploy and adds more options to the managing physicians. Early referral to centres experienced in managing such pathology is crucial for a superior outcome. However, the rarity of RPF precludes an RCT. Therefore, we recommend further studies to investigate and establish the long-term effectiveness of endovascular repair in RPF.

## Data Availability

The raw data supporting the conclusions of this article will be made available by the authors, without undue reservation.
